# Evaluating synthetic CT from deformable registration in directed adaptive radiotherapy for head and neck cancer

**DOI:** 10.1002/acm2.70732

**Published:** 2026-08-11

**Authors:** Jessica Lye, Kartik Kumar, Benjamin Harris, Jarrod Prohasky, Leah McDermott, Simon Goodall, Jothy Selvaraj, Lauren Natoli, Morikatsu Wada, Sweet Ping Ng

**Affiliations:** ^1^ Department of Radiation Oncology Olivia Newton John Cancer Wellness and Research Centre Austin Health Melbourne Australia; ^2^ RMIT University Melbourne Australia; ^3^ Ballarat Austin Radiation Oncology Centre Austin Health Ballarat Australia; ^4^ Radiation Oncology Alfred Health Melbourne Australia; ^5^ Genesis Cancer Care Alexandria Australia; ^6^ School of Cancer Medicine La Trobe University Melbourne Australia; ^7^ University of Melbourne Melbourne Australia

**Keywords:** adaptive radiotherapy, deformable registration, head and neck cancer, synthetic CT

## Abstract

**Background and purposel:**

Anatomical changes over the many weeks of radiotherapy for Head and Neck cancer patients can compromise the original treatment plan, potentially increase the risk of side effects and impact outcomes. It is difficult to decide, based on daily imaging alone, which patients will most benefit from adapted treatment plans. This work presents an automated approach to evidence‐based assessment of treatment plan robustness to anatomy changes.

**Methods:**

Directed Adaptive Radiotherapy (DART) generates a synthetic CT for calculating dose changes in the treatment planning system (TPS). The process first enhances the CBCT, then uses a double fusion to insert the smaller field‐of‐view CBCT into the larger CT. The double fusion captures both neck tilt and weight loss changes, to accurately capture the position of the head and related critical organs. The deformed CT and contours are sent to TPS for recalculation of the original plan and quantify dose changes. A retrospective study was undertaken using 12 H&N patients who had a replanning CT during their treatment. The quality of the dose distributions calculated on synthetic CT and deformed contours were evaluated, along with the sensitivity and specificity of the synthetic CT as a predictor for adaptation.

**Results:**

The contour and dose comparison between synthetic CT and clinical replan CT showed good agreement. The average Dice Similarity Coefficient (DSC) was > 80% for all contours, except for the sub‐mandibular (> 75%) and excluding cochlea. The average mean distance‐to‐agreement (MDA) was < 2 mm, and all MDAs were < 3 mm. The average DVH differences were within 1%, and all dose differences were within 3%. The total gamma passing rates were > 95%. Both the sensitivity and specificity of the synthetic CT for predicting the need for adaptation were 92%, based on a tolerance of 2 Gy change for both target coverage and OAR dose constraint limits.

**Conclusions:**

By quantifying the impact of anatomical changes on dose distributions, the DART process helps determine whether a patient would benefit from adaptive replanning. The DART method improves efficiency by reducing unnecessary imaging and replanning when the treatment remains robust against anatomy changes.

## 1 INTRODUCTION

Patients with head and neck cancers (H&N) often experience two types of positional changes that affect their treatment over the many weeks of their extensive radiotherapy schedule,^1^, ^2^ namely anatomical changes and posture changes. The latter is mostly eliminated with online imaging and correction of patient position, moments prior to treatment. The former, anatomical change is more difficult to account for with a simple set up correction. A study of treatment portal images catalogued a range of anatomical changes that altered the transmission of treatment beams by more than 5%, in ∼37% of 40 H&N patients.^3^ Changes due to weight loss or tumour shrinkage can impact the planned dose delivered with unwanted consequences when the dose is increased to organs at risk (OAR) or reduced to the target.^4^, ^5^, ^6^, ^7^

Since the advent of online imaging in the early 2000s, there has been considerable interest in developing offline treatment plan adaption strategies that respond to clinically significant anatomical changes.^8^ The aim of treatment adaptation is to reduce the incidence and severity of adverse side effects and improve local disease control. Several studies have demonstrated improved local control of disease with adaptive radiotherapy.^9^, ^10^ Radiotherapy for H&N is associated with a high risk of side effects due to the proximity of critical structures such as the salivary glands, pharyngeal musculature, spinal cord and optic structures. Common toxicities include mucositis, dermatitis, xerostomia, dysphagia and odynophagia, which significantly impact patient quality of life and may lead to treatment interruptions.^4^, ^5^ Anatomical changes can impact the dose delivered to sensitive organs and increase the risk of toxicities, such as the change in parotid volume by up to 40% and parotid migration towards the midline during radiotherapy often associated with risk of xerostomia.^11^, ^12^, ^13^, ^14^, ^15^ Several studies have shown the potential benefit of adaptation to reduce the risk of xerostomia and dysphagia.^16^, ^17^, ^18^, ^19^, ^20^, ^21^

One of the biggest obstacles to offline adaptive radiotherapy is the time taken to rescan, recontour and replan patient treatments. Tools to define patient selection for ART are lacking, often based on experience of treating physicians. There are few guidelines on clinical indicators that signify when or how often plans should be adapted.^22^, ^23^ As reviewed by Nuyts *et al.* there are important questions to answer regarding patient selection and ideal timing and frequency of ART.^24^Most clinically relevant anatomical changes occur during the first half of treatment, so many centres recommend replanning patients at a fixed time point.^18^, ^25^ Heukelom *et al.* tested the predictive power of the normal tissue complication probability and treatment adaptation, raising the importance of systematic approaches to ART to reduce inter‐observer variability.^22^

Several technical challenges must be overcome to generate high quality synthetic CTs from CBCT images for head and neck cancer. One of the main difficulties stems from the inherent poorer image quality of CBCT compared to conventional CT. The noise from scatter and limited soft tissue contrast leads to inaccurate HU mapping, and the complex mix of soft tissue, bone and air cavities makes it challenging to assign bulk electron density overrides to selected structures.^14^ The limited field of view available with CBCT images is a further challenge for large head and neck treatment areas, often missing the optical and related structures superiorly and external parts of the body inferiorly. Additionally, bolus and immobilisation masks frequently cause artefacts in deformable registration when using an automated protocol. Deformation of the original CT to the CBCT anatomical structures can overcome these issues by transferring accurate electron density values from the original CT to the current anatomy, assuming the deformation algorithm can correctly spatially translate the original HU to the new geometry. This approach was used by Allen et al. and Mukherji et al. using the Velocity software,^21^, ^27^ though Mukherji et al. required an extended CBCT to address field‐of‐view limitations, at the cost of increased imaging dose. A key limitation of the single‐fusion approach used by Allen et al. is that a single rigid registration cannot simultaneously account for both neck tilt and body weight loss, which introduce geometrically distinct changes in the head and neck region.

Once a synthetic CT has been generated, a further challenge is determining appropriate dosimetric tolerances for triggering adaptation. Barragan‐Montero et al. developed a dosimetrically‐triggered ART framework for H&N cancer using a complex, iteratively refined multi‐metric tolerance model, identifying two alert levels — surveillance and immediate verification — through sensitivity/specificity analysis against clinician decisions.^30^ While effective, such approaches require significant institution‐specific calibration and may be difficult to implement broadly in routine clinical practice.

In this work we present DART (Directed Adaptive Radiotherapy), a novel workflow implemented within MIM Maestro (MIM Software Inc., Ohio, USA). MIM is a widely available commercial radiotherapy software platform that addresses the limitations of prior approaches in three major ways. Firstly, DART employs a double fusion strategy, registering the head region independently from the body to explicitly account for neck tilt and weight loss as distinct geometric changes, improving synthetic CT accuracy over single‐fusion methods. Secondly, DART incorporates a systematic pose correction step to separate residual inter‐fraction postural variation from true anatomical change, reducing false‐positive adaptation triggers. Thirdly, DART applies a single, unified ΔGy tolerance parameter across all DVH metrics. This single tolerance provides a simpler and more clinically interpretable method than multi‐metric models — with the optimal threshold determined by ROC analysis against the clinical decision to replan as the ground truth. By implementing the full workflow within MIM Maestro, DART is directly transferable to any centre using this platform, without requiring custom software development.

The specific aims of this study were to validate the method for generating a synthetic CT from a CBCT to replicate a replan CT, and to evaluate the sensitivity and specificity of the DART workflow for predicting the need for adaptive replanning in H&N cancer patients.

## 2 METHODS

### 2.1 Study population

The study consisted of the validation and development of a synthetic CT to simulate a replan CT in order to aid treatment adaptation selection. Retrospective data was collected from 12 patients treated for H&N cancer at Austin Health from 2020–2023. Treatment planning CTs (original CT) were acquired after immobilising the patients with a thermoplastic head and shoulder mask on a Siemens Somaton AS64 CT (Siemens, Erlangen, Germany). All patients were treated on a Versa HD linear accelerator (Elekta, Stockholm, Sweden) and positioned with daily cone‐beam CT acquired using the Elekta XVI on‐board imaging system (Elekta, Stockholm, Sweden). All 12 patients had their plans re‐optimised based on a mid‐treatment repeat CT (replan CT). The treating H&N radiation oncologist contoured target structures and organs at risk (OAR) on the original CT in MIM Maestro software (version 7.4.2, MIM Software Inc., Ohio, USA). The study was approved by Austin Health Ethics Committee in 2023 HREC/103713/Austin‐2023.

### 2.2 Treatment planning

Plans were designed, optimised and calculated using Monaco (v6.1, Elekta Solutions, Stockholm, Sweden). All plans used either intensity modulated radiotherapy (IMRT, 7 patients) or volumetric modulated arc therapy (VMAT, 5 patients). The patient cohort consisted of a range of H&N disease types, as shown in Table 1, including ten bilateral and two unilateral treatments. Eleven patients had three CTV contours, and one unilateral patient had one CTV. The three dose levels included: high—the primary tumour and involved lymph nodes; medium—high‐risk sub‐clinical areas adjacent to the primary tumour or involved lymph nodes at high risk of harbouring microscopic spread and low—the elective low‐risk nodal regions.

**TABLE 1:** Patient H&N treatment types.

Patient	Type	Bi/unilateral	Staging	PTVs	Replan
1	supraglottis	bilateral	T1N0M0	PTVL, PTVM, PTVH	Week 5
2	oropharynx	bilateral	T2N1M0	PTVL, PTVM, PTVH	Week 4
3	tonsillar	bilateral	T1N3bM0	PTVL, PTVM, PTVH	Week 3
4	oropharynx	bilateral	T2N2M0	PTVL, PTVM, PTVH	Week 4
5	nasopharyngeal	bilateral	T1N1M0	PTVL, PTVM, PTVH	Week 2
6	tongue	bilateral	T2N1M0	PTVL, PTVM, PTVH	Week 4
7	nasopharyngeal	bilateral	TxNxM1	PTVL, PTVM, PTVH	Week 5
8	oropharynx	bilateral	T4N1M0	PTVL, PTVM, PTVH	Week 3
9	oropharynx	bilateral	T3N0M0	PTVL, PTVM, PTVH	Week 3
10	oropharynx	bilateral	T4aN3M0	PTVL, PTVM, PTVH	Week 4
11	supraglottis	unilateral	T3N2M0	PTVL, PTVM, PTVH	Week 1
12	tonsillar	unilateral	TxN2M0	PTV	Week 5

The CTV to PTV margin for all plans was 5 mm and dose was prescribed to three levels: 70 Gy to PTVH, 63 Gy to PTVM and 56 Gy to PTVL, corresponding to the high, medium and low CTV contours. Treatments were delivered in 35 (2 Gy) fractions. The plan optimisation aim was for the 95% isodose to cover the respective PTV, with a minimum of the 90% isodose covering PTVL. OAR dose constraints followed standard QUANTEC guidelines, including parotid gland mean dose ≤26 Gy, thecal sac D_max ≤45 Gy, brain D_max ≤60 Gy, and mandible D_max ≤70 Gy. Replanning was performed from week 1 to 5, with the majority of replans occurring in weeks 3 and 4.

### 2.3 Synthetic CT generation

**FIGURE 1:**
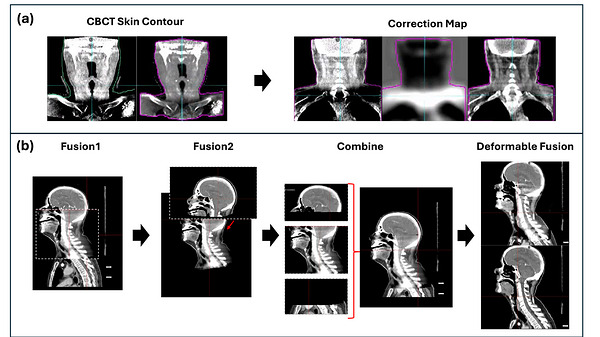
The MIM workflow showing (a) removing the bolus and enhancing the CBCT, and (b) registration and stitching to create the final synthetic CT.

The DART protocol relies on a representative synthetic CT to predict dose changes due to anatomy changes. To generate the synthetic CT, the CBCT was exported to MIM from XVI using the clinical couch shifts. The CBCT import automatically triggered the workflow using the original CT and structures previously exported from Monaco. An overview of the process is shown in Figure 1. In the first part of the workflow, bolus was removed and an enhanced CBCT was created providing a more uniform image. In the second part of the workflow there was a two‐part image registration and image stitching prior to a deformation of the original CT to generate the synthetic CT. The Fast H&N S20 CBCT settings with medium resolution were used. The main steps of the generation are described below.

### 2.4 Remove Bolus and thermoplastic mask

In the first step, the thermoplastic mask and any bolus were removed from the CBCT by removing outside the original CT skin contour (Figure 1a).

### 2.5 Find CBCT skin

The CBCT was copied and the MIM “Standardize Intensity” function was applied to correct for CBCT‐specific imaging artefacts. CBCT images are susceptible to scatter radiation, beam hardening, and photon starvation — particularly in high‐attenuation regions such as the shoulders — resulting in non‐uniform Hounsfield Unit (HU) values compared to planning CT. Standardize Intensity addresses this using a histogram‐based normalisation approach, in which the intensity histogram of the CBCT is mapped to a standard template derived from the planning CT, linearly rescaling voxel intensities to match the planning CT HU range.^32^ An external body contour was then generated to create the CBCT skin outline.

### 2.6 Correction map

Both the original CT and CBCT were masked outside the skin contour, and the voxel‐wise intensity difference between the two masked images was computed. A Gaussian smoothing kernel of 2 cm was applied to this difference image to produce a spatially smooth correction map. Unlike standard Gaussian filtering — which blurs the image directly to suppress noise — this approach smooths the residual intensity difference between modalities, creating a spatially varying correction field that avoids sharp discontinuities at structure boundaries. The correction map is then added to the CBCT to produce the “enhanced CBCT”, which has improved HU uniformity and intensity consistency with the original CT. This enhancement optimises the performance of the subsequent MIM deformable image registration algorithm.

### 2.7 Helper contours

Three contours were created for the double fusion process. Firstly, the CBCT field of view (FOV) was found by expanding CBCT skin by 10 cm first in the anterior‐posterior (AP), left‐right (LR) directions, then in superior‐inferior (SI) directions. The FOV mask was created from the intersection of the AP/LR expansion, and the SI expansion. A contraction 1 cm in SI direction avoids the end artefacts on the CBCT. Secondly the head region was identified by expanding the brainstem 10 cm LR, 15 cm A, 12 cm P, 15 cm S and 3 cm I on the CT and transferred rigidly to the CBCT. Thirdly, the overlap between the CBCT FOV and the head region was used for a contour based rigid fusion between the CBCT and the CT head region.

### 2.8 Double fusion

In the second part of the workflow, Figure 1b, the original CT was copied and cropped to the head, based on the expansion of the brainstem contour, and labelled “CT Head”. The workflow takes the enhanced CBCT and first registers it to the original CT with the shift and rotation vectors that were determined by the radiation therapists at treatment. The second registration automatically matches the CT Head to the enhanced CBCT, which allows different positions due to neck tilt to be included. This is important to maintain the correct positions of the optic and brain structures in the synthetic CT.

### 2.9 Combine

A copy of the original CT is masked for the CBCT FOV and CT Head regions and labelled “CT Body”. The three images are combined: the CT Head, CT Body and enhanced CBCT; to create a large image dataset with a full field of view.

### 2.10 Deformable Fusion

Finally, the original CT is deformed to match the anatomy of the combined image using the multi‐modality deformable image registration (DIR) algorithm, where the combined image serves as the fixed reference and the original CT as the moving, deformed image.

### 2.11 Pose Correction

Differences in patient position between imaging datasets are usually due to either posture or anatomical changes. With minimal time between CT scans, it is expected the residual differences are due to posture. Following the method used by Allen *et al.*,^21^ the synthetic CT geometry and dose calculation validation steps were performed with correction for posture. An additional calculation was performed without the correction for posture to test the effect of posture correction. The pose correction was performed in MIM using the multi‐modality DIR algorithm. The CBCT was registered to the replan CT with both datasets masked outside their respective skin contours. The replan CT was the fixed reference and the CBCT was the deformed image.

**FIGURE 2:**
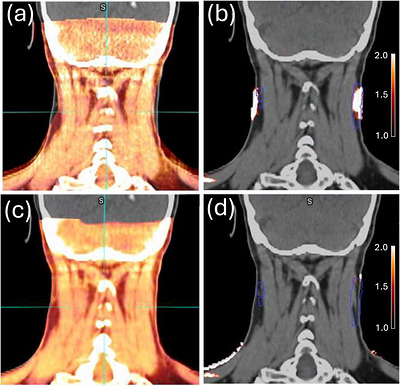
(a) Non‐pose corrected synthetic & replan CT fusion, (b) gamma index failing points (> 1) superimposed on CT for non‐pose corrected synthetic CT and replan CT dose distributions, (c) pose corrected synthetic & replan CT fusion and (d) gamma index failing points (> 1) for pose corrected synthetic CT and replan CT dose distributions.

The CBCT was pose corrected to account for the small differences in shoulder position and neck tilt between patient setup on treatment and the replan CT. This is particularly important for PTVs at the skin surface, as small positioning differences accounted for by the planning margin can artificially impact the dose assessment. The PTV contour may partially overlap with air outside the body contour, an example is shown in Figure 2. The CBCT non‐corrected and corrected coronal slice fusion are shown (a and c), as well as the gamma index of the corresponding dose differences for corrected and non‐corrected synthetic CT‐dose calculations (b and d). All steps of the validation process were repeated without CBCT pose correction, so any residual variation in patient posture added uncertainty to the dose calculation accuracy but also avoided a potential unwanted correction of an anatomical change.

### 2.12 Synthetic CT validation—contour accuracy

The quality of the synthetic CT was validated by assessing the accuracy of both the geometrical contour representation, and of the resulting adapted dose calculation for each of the study patient's datasets.

For each patient a CBCT was selected from a fraction within 3 days of the patient's replan CT. The selected CBCT was then stitched to the original CT and resulting image was deformed using the MIM Maestro workflow to form a synthetic CT (as described above). The deformation vector field was also applied to the contours, whereby the synthetic CT and deformed contours were sent to Monaco treatment planning system.

**FIGURE 3:**
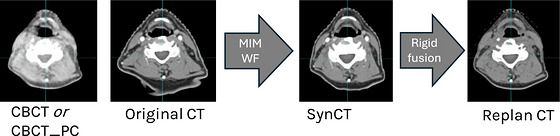
Workflow (MIM WF) to evaluate the geometric contour quality of the synthetic CT with respect to the replan CT.

The quality of the synthetic CT geometric distribution was assessed by registering each synthetic CT with the corresponding replan CT and comparing the deformed and replanned contours using the MIM software contour compare tool. The registration steps for the validation process are shown in Figure 3. The synthetic CT was generated from the original CT, then matched rigidly to the replan CT using a box‐assisted fusion algorithm that encompasses the PTV regions. It is assumed differences between synthetic CT and replan CT are due to either posture or anatomical changes, where the latter is minimal given the close acquisition times. The residual differences in posture between the treatment CBCT and the replan CT were addressed with a pose correction, as described above. The deformed contours were compared to the replan contours using the Dice similarity coefficient (DSC) and mean distance to agreement (MDA), calculated using the MIM Maestro contour comparison tool.^14^, ^20^

### 2.13 Synthetic CT validation—dose calculation accuracy

The quality of the synthetic CT dose calculation accuracy was assessed by calculating the replan on the synthetic CT and comparing the dose distributions calculated on the replan CT and synthetic CT. Following rigid registration to the replan CT (as shown in Figure 3), the synthetic CT was sent to Monaco for dose calculation using the replan contours. Two methods were used to compare the dose distributions. The first was dose‐volume histogram (DVH) metrics for target coverage and OAR sparing. The contours and DVH‐metrics used for the study are presented in Table 2.

**TABLE 2:** The dose‐volume histogram (DVH) metrics used to compare the synthetic and replanned CT dose distributions.

Contour	DVH‐metric
Brainstem	D_0.035_
Cochlea_L	D_mean_
Cochlea_R	D_mean_
Mandible	D_0.035_
Oral Cavity	D_mean_
Parotid_L	D_mean_
Parotid_R	D_mean_
Brainstem	D_0.035_
Submand_L	D_mean_
Submand_R	D_mean_
Thecal Sac	D_0.035_
PTV/CTV_H	D_2_, D_95_, D_98%_
PTV/CTV_M	D_2_, D_95_, D_98%_
PTV/CTV_L	D_2_, D_95_, D_98%_

The second method of dose comparison was a gamma analysis, both per contour and for the total clinical dose volume. Criteria for comparison were 2% local dose difference, 2 mm distance‐to‐agreement and above 10% dose threshold. A more stringent local dose difference was used to increase the sensitivity.^28^, ^29^

### 2.14 DART sensitivity and specificity

**FIGURE 4:**
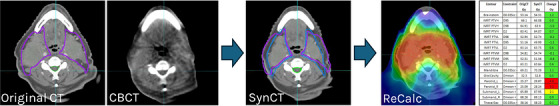
Process overview for directed ART (DART)—automatic selection assistance for offline adaptation. The original planned structures (purple) and those generated from the deformed vector field of the CBCT (blue) are shown on the original and synthetic CTs.

The DART process begins with the decision to adapt. Key to the success of this tool is setting appropriate “traffic light” tolerances that have high sensitivity and specificity. An overview of the DART process is shown in Figure 4. A re‐calculation script was used to automatically generate a new plan and a traffic light‐style dose‐volume metric report is generated, identifying when preset dose coverage or OAR dose tolerances levels are exceeded.

**FIGURE 5:**
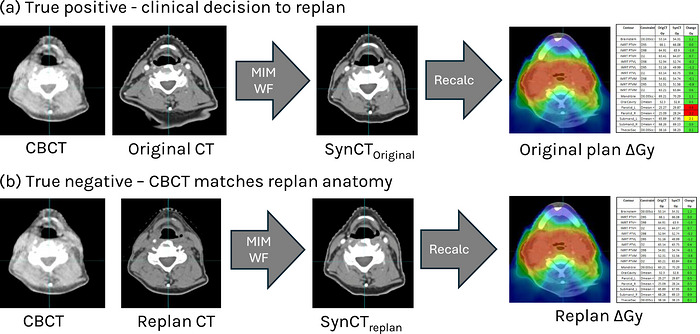
(a) shows the DART workflow to assess true positive (b) shows the DART workflow to assess true negative.

To assess the sensitivity of the adaptation selection result, the DART process was run as shown in Figure 5a. The dose changes found in the synthetic CT with respect to the original treatment plan were quantified and compared to the clinical decision to replan. To evaluate specificity limitations due to inter‐fraction changes in posture, the DART process used the replan CT instead of the original CT to create the synthetic CT, as shown in Figure 5b. The dose changes found in the synthetic CT with respect to the replan treatment plan were quantified. As the CBCT and replan CT were taken within three days of each other, only residual postural changes should impact the dose changes.

Two synthetic CTs were generated for each patient: a synthetic CT derived from the original planning CT (SynCT_original), for which dose changes were assessed using the original treatment plan; and a synthetic CT derived from the replan CT (SynCT_replan), for which dose changes were assessed using the replan treatment plan. The clinical decision to replan was used as the ground truth; since all 12 patients received a mid‐treatment replan, adaptation was considered clinically warranted for all. DART's predicted need for adaptation, based on whether the DVH metric change exceeded the ΔGy threshold, was evaluated against this ground truth as follows:

**True positive (TP)**: DART (SynCT_original) correctly flags the need for adaptation, consistent with the clinical decision to replan.
**False negative (FN)**: DART (SynCT_original) fails to flag the need for adaptation, despite the clinical decision to replan.
**True negative (TN)**: DART (SynCT_replan) correctly does not flag the need for adaptation, as no significant anatomical change exists between the replan CT and CBCT.
**False positive (FP)**: DART (SynCT_replan) incorrectly flags the need for adaptation, despite no significant anatomical change between the replan CT and CBCT.

A receiver operating characteristic (ROC) plot was used to determine the optimum tolerances for adaptation selection criteria. If the tolerances are too strict, the minor inter‐fraction anatomy and posture changes may cause DART to recommend an unnecessary replan, leading to poor specificity. On the other hand, larger tolerances risk missing clinically relevant changes in dose delivered, leading to poor sensitivity. A simple single parameter of change in dose ΔGy was used for all DVH metric planning goals and target constraints. If target coverage was reduced by ΔGy, or an OAR dose constraint was exceeded by ΔGy, the patient would be selected for adaptive replanning.

## 3 RESULTS

### 3.1 Synthetic CT validation—contour accuracy

**FIGURE 6:**
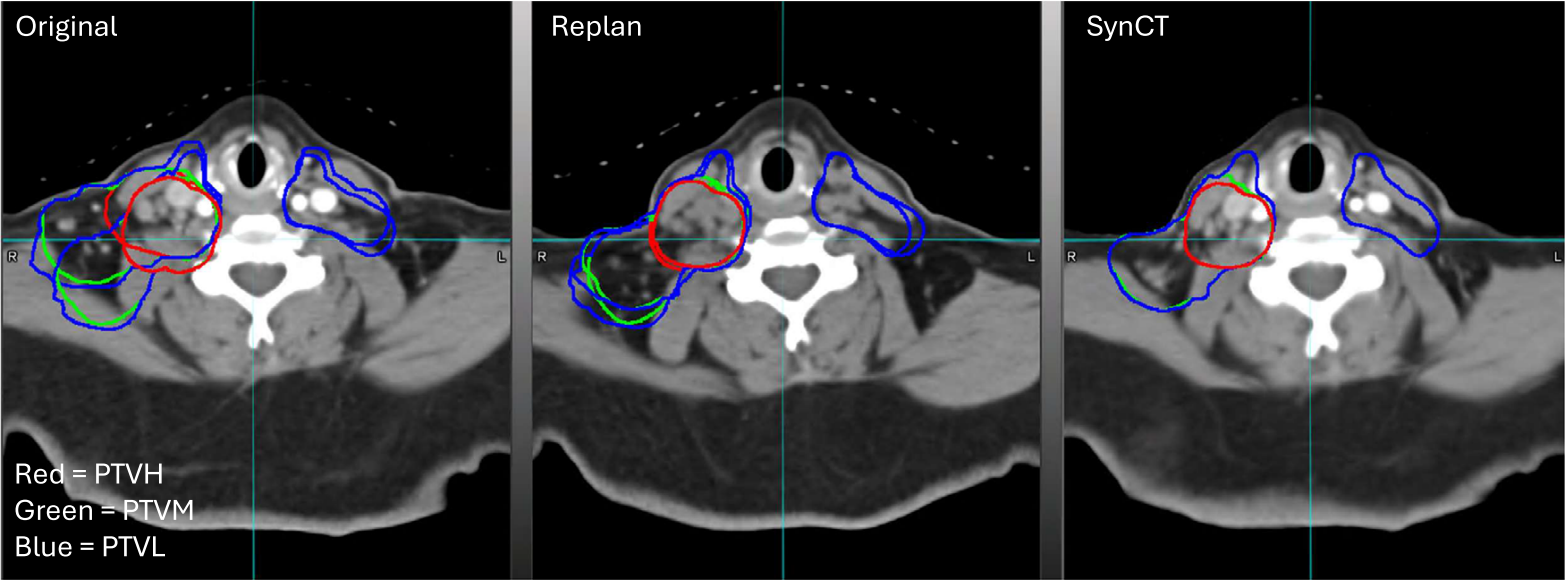
Example of synthetic CT quality assessment comparing the deformed contours to the replan contours for Patient #3. The deformed contours are overlaid with the original contours in left panel, and with the replan contours in middle panel.

**FIGURE 7:**
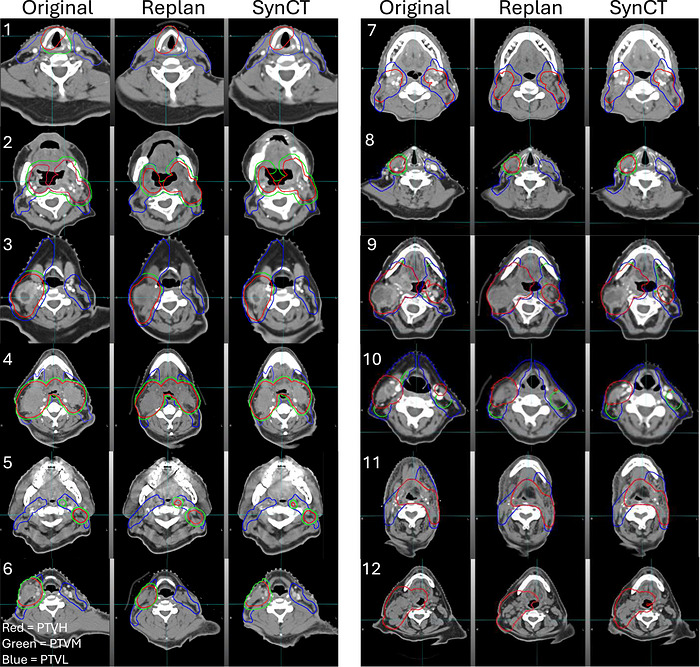
Example slice for each patient showing PTVH, PTVM, and PTVL, showing synthetic CT quality compared to replan and original CT.

For each patient, the original CT was deformed to the stitched combined image (CT Head, CT Body and enhanced CBCT) as the fixed reference, producing the synthetic CT used for all subsequent contour and dose comparisons. An example of the retrospective synthetic CT quality assessment is shown in Figure 6 comparing the deformed contours to the replan contours. For each patient a representative slice of the Original CT, Replan CT and Synthetic CT containing the PTVH is shown in Figure 7. Many of the patients displayed large changes in the neck and shoulder region due to weight loss, and some also showed changes due to tumour shrinkage.

**FIGURE 8:**
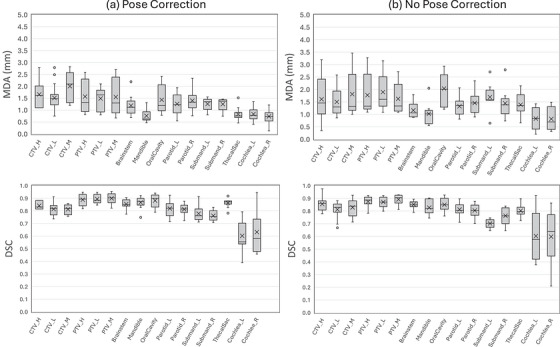
The mean distance‐to‐agreement (MDA) and dice similarity coefficient (DSC) for target structures and organs at risk (OAR) (a) with pose correction and (b) without pose correction.

The results for both target and OAR contours are summarized in Figure 8. The results for both target and OAR contours are summarized in Figure 7. Contour agreement was assessed against literature‐reported acceptability thresholds of DSC > 0.75–0.8 and MDA < 2–3 mm.^14^, ^20^ The DSC and MDA comparing synthetic and replanned OAR and target structures showed good agreement for the pose corrected analysis. For the cochlea structures, only the MDA is considered a robust metric due to the unreliability of DSC for very small contours with insufficient data points. The average DSC for the targets was > 80%, and all target DSC were > 75% for the pose corrected analysis. The average DSC was > 80% for all OARs, except sub‐mandibular with an average DSC > 75% and cochlea > 60%. In the pose corrected data, all OAR DSC were > 70%, except cochlea with > 60%, and the average MDA was ≤ 2 mm, and all MDA results were < 3 mm. Without the pose correction, slightly larger differences were observed, as expected due to posture differences between the replan CT and the treatment CBCT.

### 3.2 Synthetic CT validation—dose calculation accuracy

**FIGURE 9:**
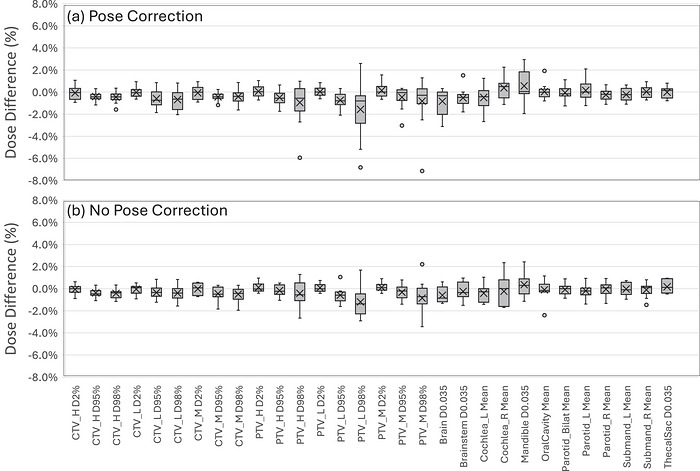
Shows the differences in DVH metrics per contour between the synthetic and replanned CT dose distributions for target structures and organs at risk (a) with synthetic CT pose correction, and (b) without synthetic CT pose correction.

A summary of the comparison of synthetic and replanned dose distributions by DVH‐metric is shown in Figure 9, including the changes in target dose results both with and without pose correction. Without pose correction (Figure 9b), the CTV DVH‐metric differences, D2% are all within 2%, and the average differences were all within 1%. The DVH OAR differences were within 3% and the average differences within 1%. The PTV D98% without pose correction showed two patients with outliers > 5%. This was due to the treatment setup variation causing some posture differences between CBCT and replan CT, where the replan contour fell outside the skin surface. Once pose correction was included (Figure 9a), the PTV dose calculation errors due to contours falling outside the skin contour were compensated, while also correcting any residual anatomy changes. The pose corrected dose differences between the synthetic and replanned dose distributions per contour, as determined from DVH metrics, are all within 3%, except for one patient with a PTV_M D_98%_ difference of 3.4%.

**FIGURE 10:**
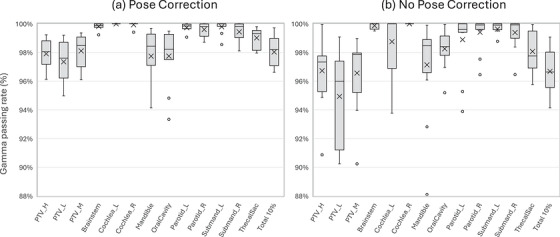
Shows the passing rates for the contour limited gamma (2%/2 mm, 10% threshold) for synthetic CT created with (a) pose corrected synthetic CT and (b) without pose corrected synthetic CT.

A summary of the gamma analysis comparing synthetic and replanned dose distributions, based on 2% and 2 mm criteria, is shown in Figure 10. The gamma pass rate across the total dose distribution within the patient body contour, restricted to voxels receiving more than 10% of the prescribed dose, was ≥94% without pose corrected (Figure 10b), and ≥95% with pose correction (Figure 10a). The gamma pass rates were also assessed for target and organ contours separately. All gamma pass rates per contour were > 90% with pose correction. Without the pose correction, for one patient the mandible result was 88%, due to neck tilt posture differences between the CBCT and the replan CT.

### 3.3 Directed ART sensitivity and specificity

The largest change in dose metrics (ΔGy) for both original plan comparison and replan comparison with the synthetic CT were determined for each patient. Only a reduction in dose for targets or increase in dose for OARs was considered, if dose constraints were exceeded. For the replan comparison, the CBCT is well matched to the replan CT anatomy, with only one patient where a reduction in PTV D98% exceeded 2 Gy. For the original plan comparison, where the CBCT shows significant differences due to anatomical changes such as weight loss, only one patient had ΔGy less than 2 Gy across all contours. For a 70 Gy prescription, 2 Gy corresponds to approximately 3% for PTV planning goals, 4% for brainstem and thecal sac constraints, 5% for submandibular glands, and 8% for parotid glands.

**FIGURE 11:**
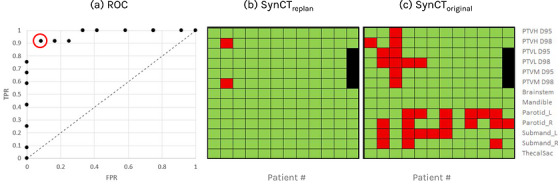
(a) Shows the ROC curve, with optimum threshold at 2 Gy circled in red, and the dashed line showing random prediction. (b) and (c) The individual Pass (green), Fail (red), N/A(black) for each contour at 2 Gy for the replan and original comparison, respectively.

Using an ROC approach, as shown in Figure 11 (a), the Δ2Gy threshold (circled) is the closest to the optimal upper left‐hand corner, with a sensitivity of 92% and specificity of 92%. Figure 11 (b) and (c) show the individual Pass/Fail for each contour at 2 Gy for the replan and original comparison respectively. The one patient who flagged for adaptation on the replan comparison had a drop in PTVH and PTVM D_98%_ of 2.6 Gy and 2.7 Gy, respectively. This patient was extremely thin, with a PTV extending to the surface of the skin, and the plan appeared very sensitive to small changes in patient position. For one patient, changes in dose to the original plan were quite robust despite significant weight loss, and may not have required a replan CT. Another two patients had smaller dose changes, one with a reduction of PTV D_98%_ of 2.1 Gy, and another with parotid mean increasing by 2.4 Gy. Is possible that these patients did not need their replan CT at week 5 of treatment. The DART process was run with CBCTs for fractions in the 1^st^ week of treatment, whereby six of the twelve patients demonstrated significant weight loss and showed ΔGy > 2 Gy. This dose change before completing 1 week of treatment suggests they may have benefited from a replan during their treatment at an earlier time than the one they received.

## 4 DISCUSSION AND CONCLUSIONS

The findings from this exploratory study support the feasibility of synthetic CBCT‐based semi‐automated directed adaptive radiotherapy (DART) for patients undergoing radiotherapy for head and neck cancer. Anatomical changes during treatment, such as tumour regression weight loss, and consistent positional changes, can lead to clinically significant deviations in planned dose distribution. What is difficult to predict is plan robustness to these anatomical changes to accurately select patients who would benefit from a replan, underscoring the importance of accurately assessing the dosimetric robustness of the treatment plan during the course of therapy.

The semi‐automated workflow developed in this study addresses several persistent barriers to widespread ART implementation. These include time, resource burden and the challenge of reliably predicting when a patient will benefit most from replanning. With the use of an enhanced CBCT combined with double fusion and deformable registration techniques, the workflow allows users to generate high‐quality synthetic CTs. The generated CTs demonstrated agreement with clinical replan CTs in terms of both geometry and calculated dose. The DSC and MDA met clinical acceptability thresholds for both targets and organs assessed against literature‐reported acceptability thresholds of DSC > 0.75–0.8 and MDA < 2–3 mm.^14^, ^20^ The exception was the DSC for cochlea structures where lower agreement was observed, however the limitation of DSC on very small contours with insufficient data points is well known ^20^ and MDA is more appropriate. Cochlea MDA was within 1.5 mm in all cases. DVH analysis and gamma comparisons indicated that recalculated doses on synthetic CTs were consistent with replan doses, particularly when pose correction was applied to account for setup variability. It was noted that the changes observed between targets and OARs will affect the dose differently and that the pose correction will compensate for some of the observed anatomical changes between planning and replanning CTs.^8^

Across the limited patient cohort, this study quantified the predictive performance of the DART process using a clinically relevant threshold of 2 Gy deviation for key DVH metrics. The use of ROC analysis allowed the determination of optimal sensitivity and specificity, suggesting DART could serve as an evidence‐based trigger for adaptation. This is consistent with the work of Allen et al, where a 2.5 Gy tolerance was identified using a single fusion approach and Velocity for deformation.^21^ In the study by Barragan‐Montero *et al.* a more complex model of dosimetry change tolerances were developed, which was then iteratively improved with a sensitivity/specificity analysis compared to clinician decision.^30^ They identified two levels, orange (surveillance suggested) or red flag (immediate verification required).

It is important to note that the DART trigger for adaptation is not based solely on PTV coverage metrics. As shown in Table 2, the DVH assessment encompasses a set of both target and OAR metrics, including mean dose to the parotid and submandibular glands, and maximum dose to the brainstem, mandible and thecal sac. Consistent with the known patterns of anatomical change during H&N radiotherapy, OAR dose changes — particularly to the salivary glands — were drivers of adaptation flags in the majority of patients in this cohort. Four out of the twelve patients also had reduction in D98 to the elective low dose target region, and only two patients in this cohort had significant changes in the high dose targets. Conformity‐related changes, such as increased low‐dose spread to adjacent OARs resulting from body contour reduction, are captured through assessment of a range of DVH metrics, and therefore the DART method is not limited to tumour coverage assessment alone.

It should be noted that while this study included a heterogenous sample of head and neck cancer sub‐types, it was a small cohort of patients and further validation in larger, prospective cohorts is needed to confirm the cohort specific observations. Additional clinical assessment of the contours and dosimetry generated in the workflow should be included in further studies, identifying any limitations and individual patient replanning considerations.

The DART process could be used in response to changes observed in daily imaging, such as when skin contour changes are approaching a tolerance of 1 cm, or as a daily surveillance tool for all fractions. The accumulated data from automated DART has potential in machine learning models to enhance decision‐making, predicting which patients are most likely to benefit from adaptation by analysing trends in anatomical changes and tumour response, such as in Rachi *et al*.^31^ Machine learning algorithms can assist in determining the optimal timing for replanning, based on dose accumulation and anatomical thresholds, thereby reducing unnecessary interventions.

In conclusion, ART should be considered an essential component of modern radiotherapy protocols for Head and Neck cancer, to optimise patient outcomes while minimising treatment‐related toxicities, and automated solutions are needed The DART workflow shows potential as an automated dose assessment using synthetic CTs derived from daily CBCT imaging for efficient, evidence‐based identification of patients who warrant treatment adaptation, improving precision without unnecessarily burdening clinical resources.

## AUTHOR CONTRIBUTIONS

Jessica Lye—Project Conception, Protocol development, workflow development, data analysis, primary manuscript writer. Kartik Kumar—Protocol development and data analysis. Benjamin Harris—Support in workflow development. Jarrod Prohasky—Protocol development and workflow testing. Leah McDermott—Manuscript preparation and analysis refinement. Simon Goodall—Collaborator, scripting support, and workflow refinement. Jothy Selvaraj—Scripting support. Lauren Natoli—Initial protocol development and workflow testing. Morikatsu Wada—Protocol development and clinical advisor. Sweet Ping Ng—Protocol development, clinical advisor, manuscript refinement.

## CONFLICT OF INTEREST STATEMENT

The authors declare no conflicts of interest.

## ETHICAL APPROVAL

The study was approved by Austin Health Ethics Committee in 2023 HREC/103713/Austin‐2023.
